# A modified surgical approach of the hip in children: is it safe and reliable in patients with developmental hip dysplasia?

**DOI:** 10.1007/s11832-015-0659-7

**Published:** 2015-06-10

**Authors:** Yusuf Iyetin, Ismail Turkmen, Yavuz Saglam, Mehmet Akif Akcal, Koray Unay, Bahattin Unsac

**Affiliations:** Department of Orthopedics and Traumatology, Istanbul Pendik Bolge Hospital, Istanbul, Turkey; Department of Orthopedics and Traumatology, Beykoz State Hospital, Istanbul, Turkey; Department of Orthopedics and Traumatology, Bahcelievler State Hospital, Atakoy 4. Kisim, TO 109, Daire: 18, Bakirkoy, Istanbul, Turkey; Department of Orthopedics and Traumatology, Antalya Atatürk State Hospital, Istanbul, Turkey; Department of Orthopedics and Traumatology, Istanbul Medeniyet University Göztepe Training and Research Hospital, Istanbul, Turkey

**Keywords:** Surgical approach, Medial open reduction, Developmental hip dysplasia, Avascular necrosis

## Abstract

**Purpose:**

Treatment is easier and complications are less likely to occur if developmental dysplasia of the hip (DDH) is diagnosed early. In this study, we examined the early results of open reduction using a medial approach which we had modified for DDH and analyzed the success of this technique and the associated complication rates, with a focus on avascular necrosis (AVN).

**Methods:**

This is an Institutional Review Board-approved retrospective review of all patients diagnosed with DDH and treated with a modified medial approach at a single institution from July 1999 to December 2010. The patients' charts were analyzed for clinical and radiographic features.

**Results:**

Fifty-five hips of 41 patients, all of whom were treated by open reduction using a modified medial approach due to DDH, were evaluated retrospectively. The mean age of the patients at surgery was 19 (range 11–28) months, and the average follow-up was 5.5 (range 3–9.5) years. AVN was the most important complication in terms of radiological outcomes as assessed according to the Kalamchi–McEwen classification. Radiologic results were excellent or good in 51 hips (92.7 %) and fair–plus in four (7.3 %). Type 1 temporary AVN was detected in only two hips (3.6 %), and the lesions had disappeared completely in the final control graphs of these two patients. A secondary intervention was needed for two hips (3.6 %) of the same patients who were operated on due to bilateral DDH. No other complications, such as infection, re-dislocation, or subluxation, were seen in the operated patients.

**Conclusions:**

We believe that treatment for DDH using a modified medial approach during early childhood is an effective and reliable method with low AVN rates. As shown here, this method achieves great success in radiological and clinical outcomes after a minimum 3-year follow-up.

## Introduction

Developmental dysplasia of the hip (DDH) is one of the most common musculoskeletal problems in newborns [1]. The incidence of DDH varies according to geographical region and ethnicity, ranging from 0.5 to 30 % in published reports [2]. Treatment is easier and complications are less likely to occur when DDH is diagnosed early [3, 4]. In newborns and infants aged <6 months, the initial treatment method is a reduction of the hip joint using several braces [5]. Closed or open reduction of the hip joint is recommended in cases where treatment with orthosis has failed [6]. Open reduction of DDH using the medial approach is one of the most effective surgical treatment methods during early childhood [7]. Surgical treatment with open reduction through the medial approach was first described by Ludloff in 1908 [8]. In 1971, anteromedial modifications were reported by Mau et al. [9], and posteromedial modifications were reported in 1973 by Ferguson [10].

One of the problems that can be observed during the medial approach treatment is avascular necrosis (AVN) of the femoral head. Although a true evaluation of this condition requires long-term follow-up into adulthood, it is essential to pay attention to the factors (limited interventional surgery, appropriate reduction, and immobilization of the proper position) that are influential in preventing the problem [7].

Different methods of approach, immobilization, and follow-up have been suggested for a medial intervention. At our clinic we have modified the medial intervention technique and achieved reduced AVN rates. In this study, we examined the early results of open reduction using a medial approach that we modified for DDH and analyzed the success of this technique and the associated complication rates, with a focus on AVN.

## Patients and methods

This is an Institutional Review Board-approved retrospective review of all patients diagnosed with DDH and treated with a modified medial approach at a single institution from July 1999 to December 2010. Patients with neuromuscular and teratological hip dysplasia and patients who were not followed up were excluded. Surgeries were performed by a single senior pediatric orthopedic surgeon. Patients who had a minimum period of 3 years of follow-up after initial surgery were included in the study. The patients’ charts were analyzed for clinical and radiographical features. Collected data included demographics, additional co-morbidities, surgical procedure, postoperative treatment, and complications.

### Evaluation of radiologic outcomes

For the radiologic evaluation, we measure the center-edge angle of Wiberg (CEA), acetabular angle of Sharp, and the articulo-trochanter distance (ATD) values of the patients during their final checkup using the follow-up criteria of Omeroglu et al. [11]. In this classification system, each radiographic measurement is divided into three subgroups and assigned a point score (0, 1, and 2) according to their previously determined values. We have also added three corrective items relating to the existence of middle/posterior acetabular deficiency, secondary operation, and resubluxation/redislocation, respectively, to the classification system. In the presence of any of these items, 1 point for each item is extracted from the total point score. A total of 5 or 6 points represents a satisfactory outcome, while a score of <5 points represents an unsatisfactory outcome [11] (Table 1). The AVN evaluation is carried out in accordance with the Kalamchi–McEwen classification (Table 2) [12].

**Table 1:** Radiographic classification system used to assess the results

Radiographic parameters	2 points	1 point	0 points
Center-edge angle of Wiberg (°) (CEA)	≥15^a^	0–14^a^	<0^a^
	≥20^b^	5–19^b^	<5^b^
Acetabular angle of sharp (°)	≤49^a^	50–55^a^	>55^a^
	≤43^b^	44–49^b^	>49^b^
Articulo-trochanter distance (mm) (ATD)	From 0 to +10^a^	From −1 to −5 and from +11 to +15^a^	Less than −5 and more than +15^a^
	From −11 to +1^b^	From −12 to −17 and from +2 to +7^b^	Less than −17 and more than +7^b^

Corrective items (–1 point for each): (1) existence of an acetabulum in which there is a considerable distance between the most lateral point of subchondral sclerosis and the most lateral point of the acetabular roof and the subchondral sclerosis is ill defined and irregular; (2) secondary procedures (closed reduction, soft tissue and/or bony surgery); (3) early redislocation or resubluxation. Total points: 6 = excellent; 5 = good; 4 = fair–plus; 3 = fair–minus; <3 = poor; 5–6 = satisfactory; ≤4 = unsatisfactory

^a^Skeletally immature hip (1 or more of the following items are still visible on the plain radiograph: triradiate cartilage, proximal femoral physis, greater trochanteric physis)

^b^Skeletally mature hip (all of the following items are not visible on the plain radiograph: triradiate cartilage, proximal femoral physis, greater trochanteric physis)

**Table 2:** Kalamchi–MacEwen classification

Groups according to Kalamchi–MacEwen classification system for AVN	Criteria for Kalamchi–MacEwen classification system for AVN [12]
Group 1	Failure of appearance of the ossific nucleus during the first year after reduction
	Broadening of the femoral neck
	Increased radiographic density followed by fragmentation
	Present of persistent stiffness after cast removal even without radiological criteria may be the earliest sign of ischemic necrosis
Group 2	Damage of the lateral aspect of the growth plate
	Radiographs show lateral physeal bridging, and a lateral metaphyseal notch or defect
	Patients in this group develop subcapital coxa valga, with a tendency to have poor acetabular coverage
Group 3	Damage of the physis with a large central defect
	A short femoral neck without varus or valgus
	Relative ‘overgrowth’ of the greater trochanter and limb-length discrepancy
Group 4	Damage to the entire femoral head and physis
	Irregular femoral head with varus, flattening, and coxa magna
	‘Overgrowth’ of the greater trochanter, limb-length inequality, and subsequent early arthritis

*AVN* avascular necrosis

### Surgical technique

**Fig. 1:**
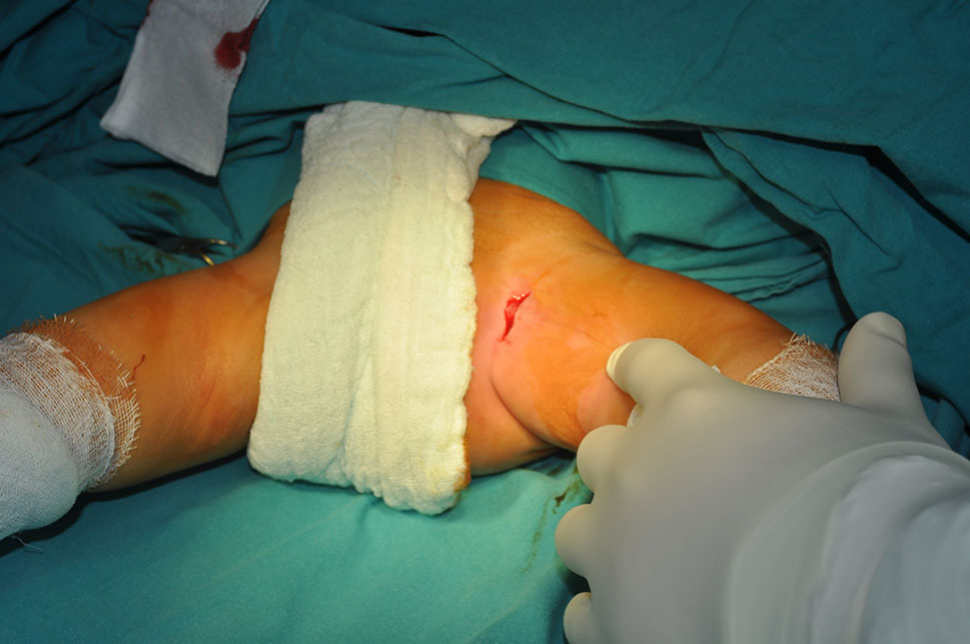
The hip is flexed 90° and abducted as wide as possible. The left knee is also flexed 90°

**Fig. 2:**
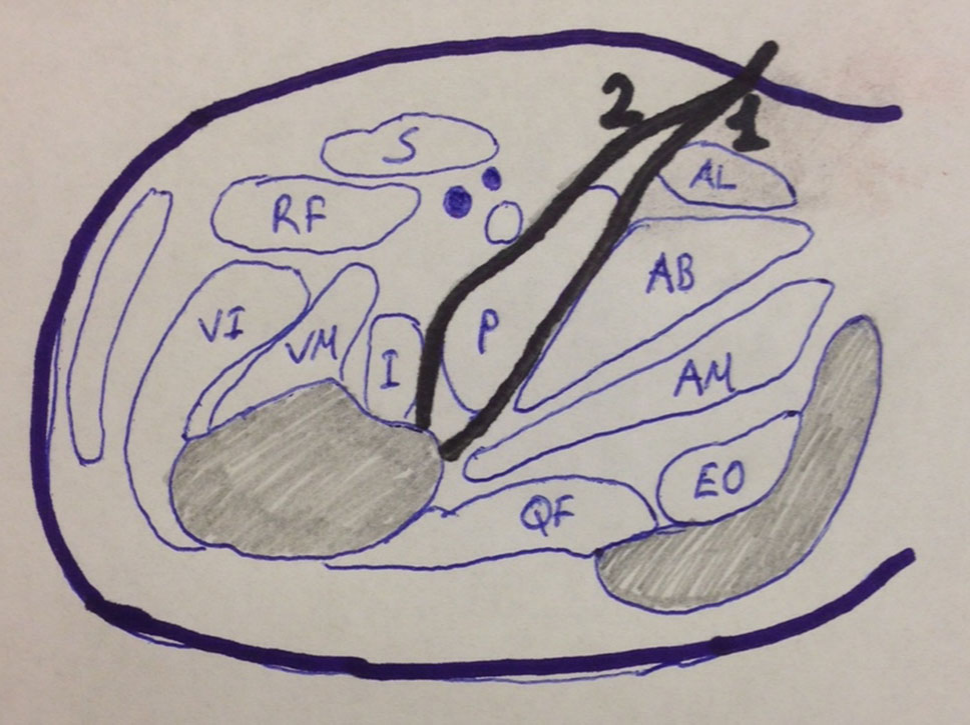
Schematic drawing of modified medial approach intervals used. Intervals: *1* was used for iliopsoas tenotomy and *2* was used for T-shaped capsulotomy, intra-articular pulvinar, ligamentum teres excision, transverse acetabular ligaman resection, and hip reduction. *AL* Adductor longus, *AB* adductor brevis, *AM* adductor magnus, *EO* obturator externus, *QF* quadratus femoris, *P* pectineus, *I* iliopsoas, *VM* vastus medialis, *VI* vastus intermedius, *RF* rectus femoris, *S* sartorius

**Fig. 3:**
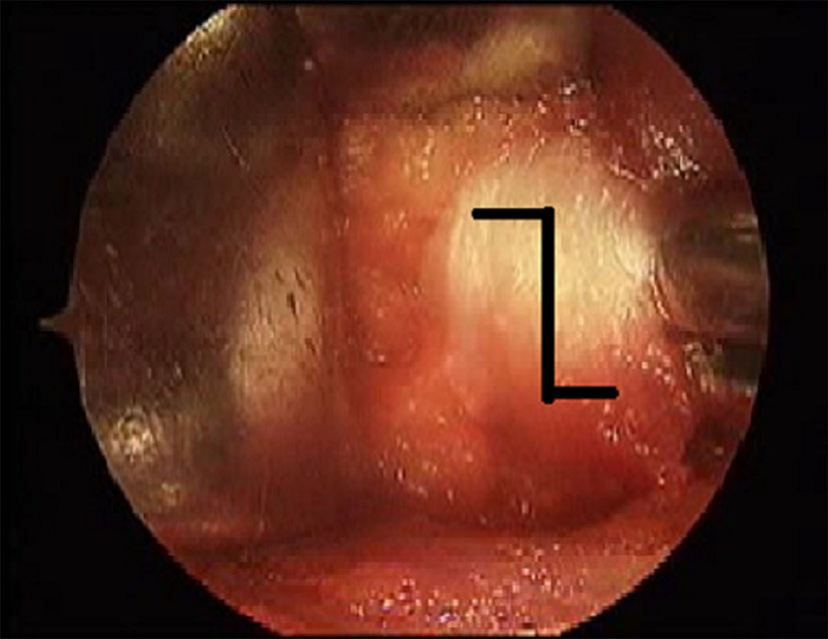
The iliopsoas tenotomy as a Z plasty

**Fig. 4:**
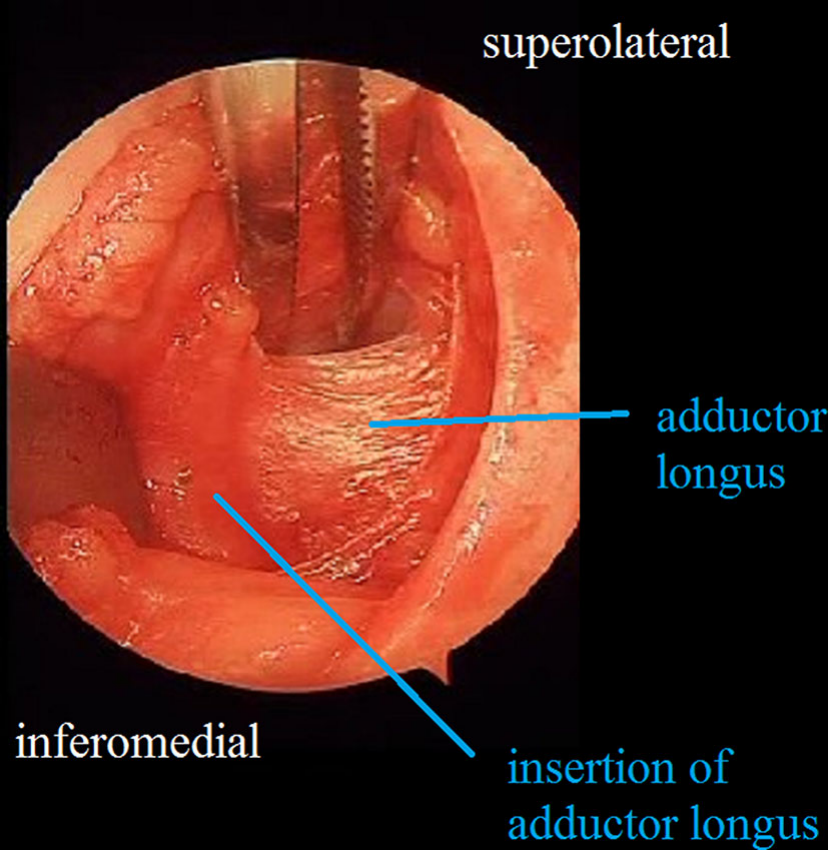
Blunt dissection in front of adductor longus

**Fig. 5:**
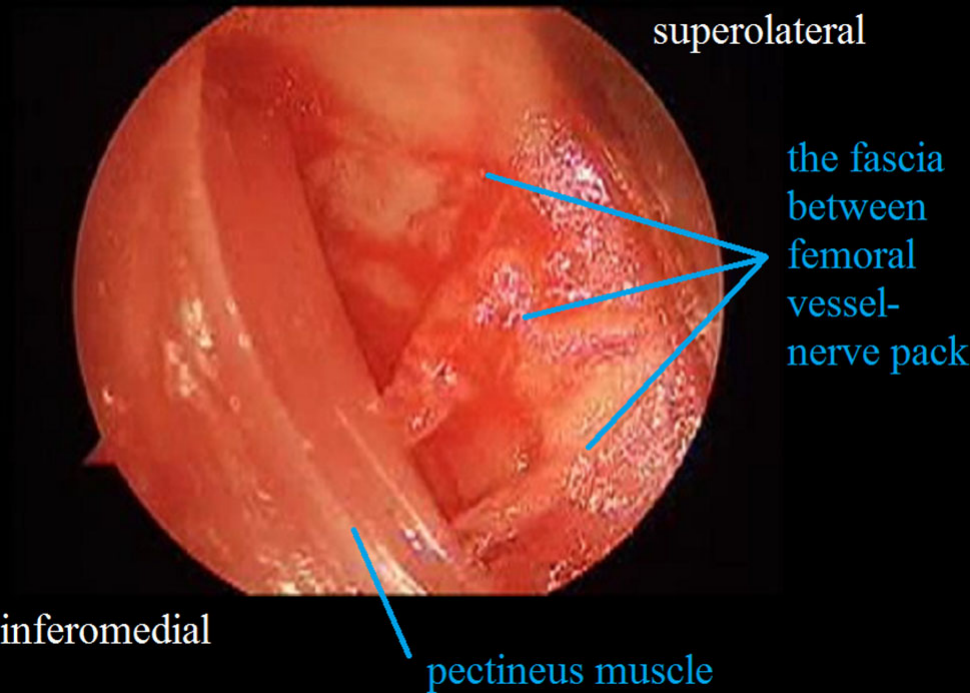
The fascia between pectineus and femoral vessel nerve pack

**Fig. 6:**
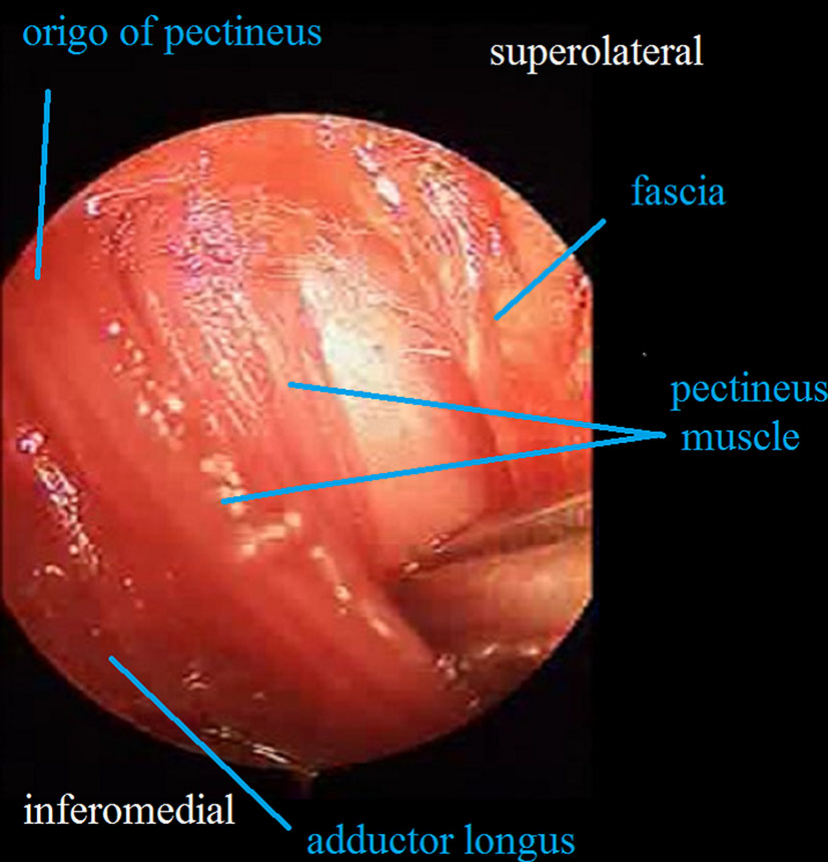
Dissection of pectineus and revealing of joint capsule

**Fig. 7:**
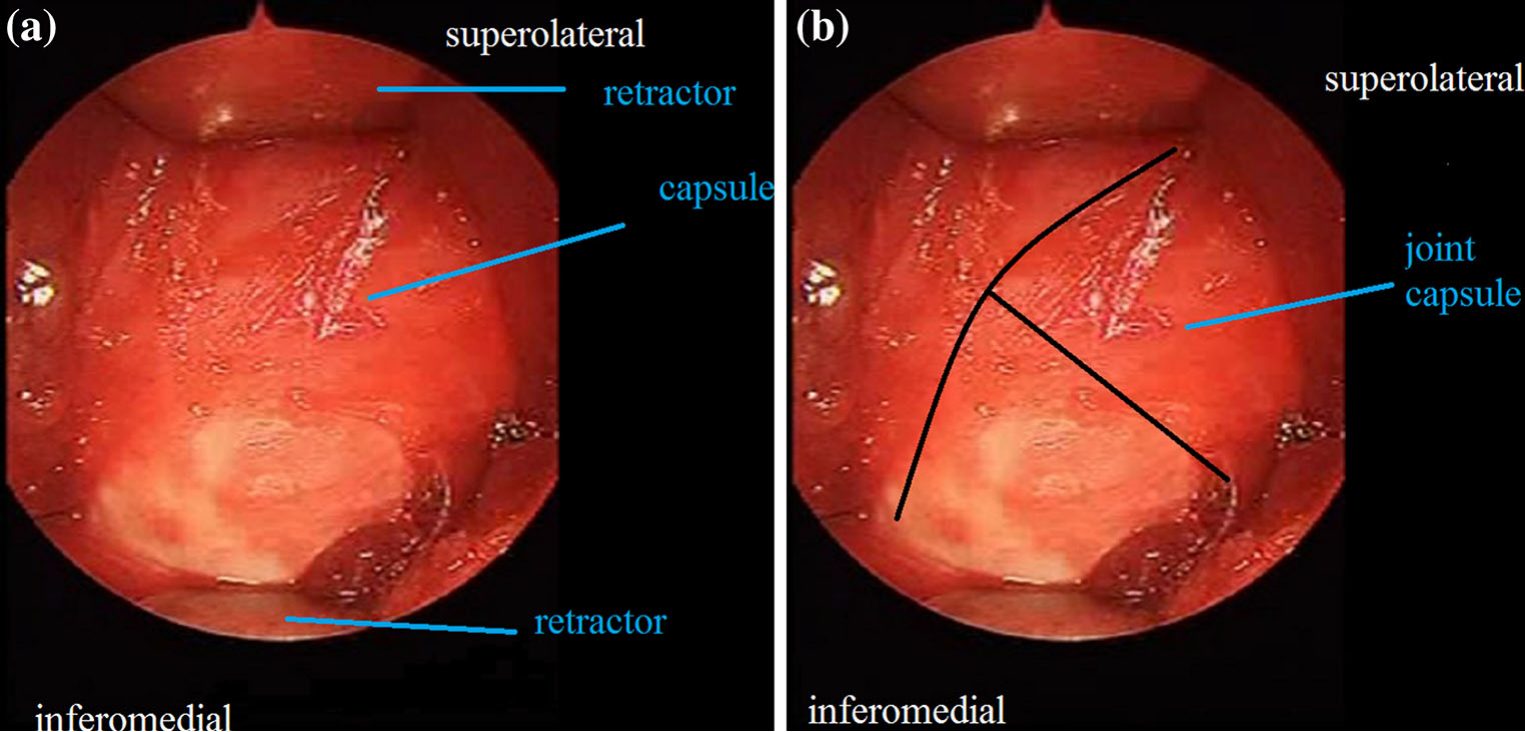
**a** Joint capsule, **b** T-shaped dissection of capsule

A transverse 3-cm skin incision is made above the inguinal crease so that the point of insertion of the adductor longus to the pubic line is centered (Fig. 1). A tenotomy is then performed at the point of insertion to the adductor longus using a cautery. The iliopsoas tenotomy is performed through the interval between the brevis and adductor longus as described by Ferguson [10] (Fig. 2). Circumflex veins in this region enter into the basicervical region of the femoral neck. The circumflex veins appear to remain proximally during the time needed to identify the point of insertion of the iliopsoas tendon. In contrast, it is safer if the iliopsoas tendon is found at the level of the trochanter minor, and the circumflex veins are not confronted. The trochanter minor is palpated with a finger; during this process it is helpful to make rotational movements of the femur. Following palpation of the trochanter minor, the interval between the adductor longus and brevis is opened with the help of retractors, and a tenotomy of the iliopsoas tendon is performed at the point of insertion as a Z plasty (Fig. 3). The capsule is then located and opened. We do not reach the capsule through the tenotomy of iliopsoas; rather, we go to the insertion point of the adductor longus and reach the capsule through the frontal pectineus muscle as described by Ludloff [8]. The dissection is done at the insertion point of the adductor longus (Fig. 4), and the pectineus muscle is revealed (Figs. 5, 6). Muscle fascia is present in the anterolateral of the pectineus muscle (Fig. 5), and this fascia serves as a guide, shielding the femoral vessel nerve pack which should not be injured. The capsule is reached deep through the pectineus and this fascia (Fig. 6). The capsule is hen dissected with the help of retractors (Fig. 7). The capsule continues to be revealed along the superolateral and inferomedial aspects of the acetabulum. A retractor is placed on the capsule under the fascia between the pectineus muscle and the femoral vessel nerve pack and the capsule dissected from below this retractor toward the superolateral aspect of the acetabulum with dissection scissors. This is one of the key points of the surgery. In the same way, another retractor is inserted to the pectineus and adductor longus over the capsule. The capsule is once again dissected toward the inferomedial to the acetabulum with dissection scissors (Fig. 7). We do not expose the obturator nerve, as both the pectineus muscle and adductor longus are excluded toward the inferomedial aspect of the acetabulum over the capsule with a retractor inserted into the anterolateral aspect of the pectineus muscle while the capsule is being revealed. The obturator nerve has anterior and posterior branches. The posterior branch is more posteromedial and more distant in comparison to the interval where we reach the capsule, as it innervates the adductor magnus and brevis. In contrast, the anterior branch moves below the adductor longus and pectineus over the adductor brevis [13]. Because we take both the pectineus and adductor longus from the anterolateral borders to the medial with the help of a retractor, the anterior branch of the obturator nerve lies within the interval entered.

**Fig. 8:**
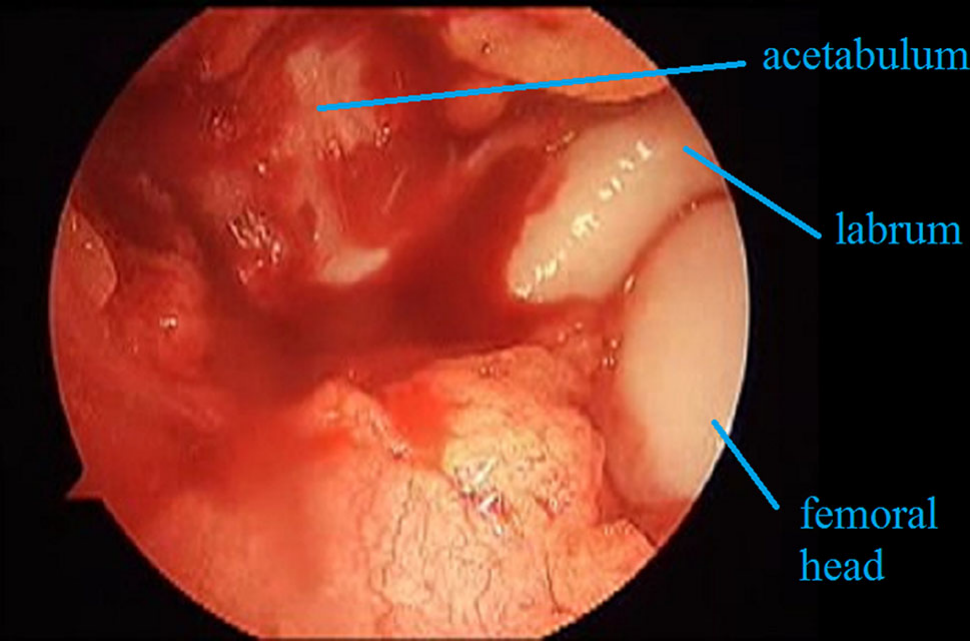
The femoral head, acetabulum and labrum before reduction

After the capsule is sufficiently dissected, it is cut in a T shape (Fig. 8). During this process, we do not encounter the circumflex veins. Enlarging the T cut on the capsule in such cases as an hourglass capsule is occasionally necessary. Under such circumstances, one needs to be careful while extending the cut at the low end of the T: if it is extended too much, the circumflex veins, which take a transverse course at the basicervical part of the femoral head, might be seen (Fig. 8).

After the capsule has been opened, the ligamentum teres and transverse acetabular ligament are excised, as well as the intra-articular pulvinar. The hip is then reduced. Capsulorrhaphy is not required. To date, none of the patients operated on using this technique have required blood transfusion. Bilateral hip dislocations are operated on in the same session. The surgery is concluded with mobilization of the hip in a spica cast in the human position.

**Fig. 9:**
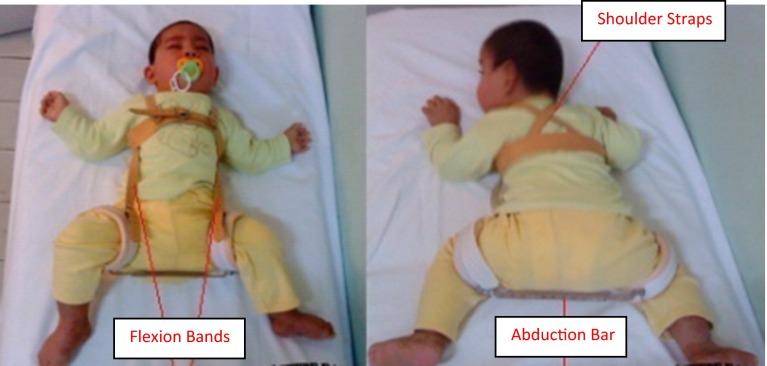
Modified Ilfeld device

A modified Ilfeld device is used following the plaster cast (Fig. 9). Among the patients included in the study group, those who were 11–18 months old at surgery were treated with plaster casting for an average of 3 months, bracing with a modified Ilfeld device for 4 months, and bracing at night only with the Ilfeld device for approximately an 2 additional months. Patients who were 18–28 months old at the time of surgery were treated with plaster casting for 3.5 months, and a modified Ilfeld device was used for 6 months initially and at night only for 2 additional months. The plaster cast was not changed in any of the patients.

## Results

Fifty-five hips of 41 patients (36 girls, 5 boys) were included in the analysis. The mean age of the patients at surgery was 19 (range 11–28) months. The average length of follow-up was 5.5 (range 3–9.5) years, and the average age when the patients last went for a check-up was 7 years, 2 months (range 5–12 years). Eleven patients had right hip DDH, 14 patients had left hip DDH, and 15 patients had bilateral DDH. None of the patients were treated with preoperative traction.

At the last follow-up, the average sharp angle was 46.4° (range 38°–55°) and the average CE angle was 27.1° (range 15°–54°). ATD values were 0–10 mm in 47 hips (85.4 %), −1 to −5 mm in five hips, and >10 mm in three hips (5.4 %).

**Fig. 10:**
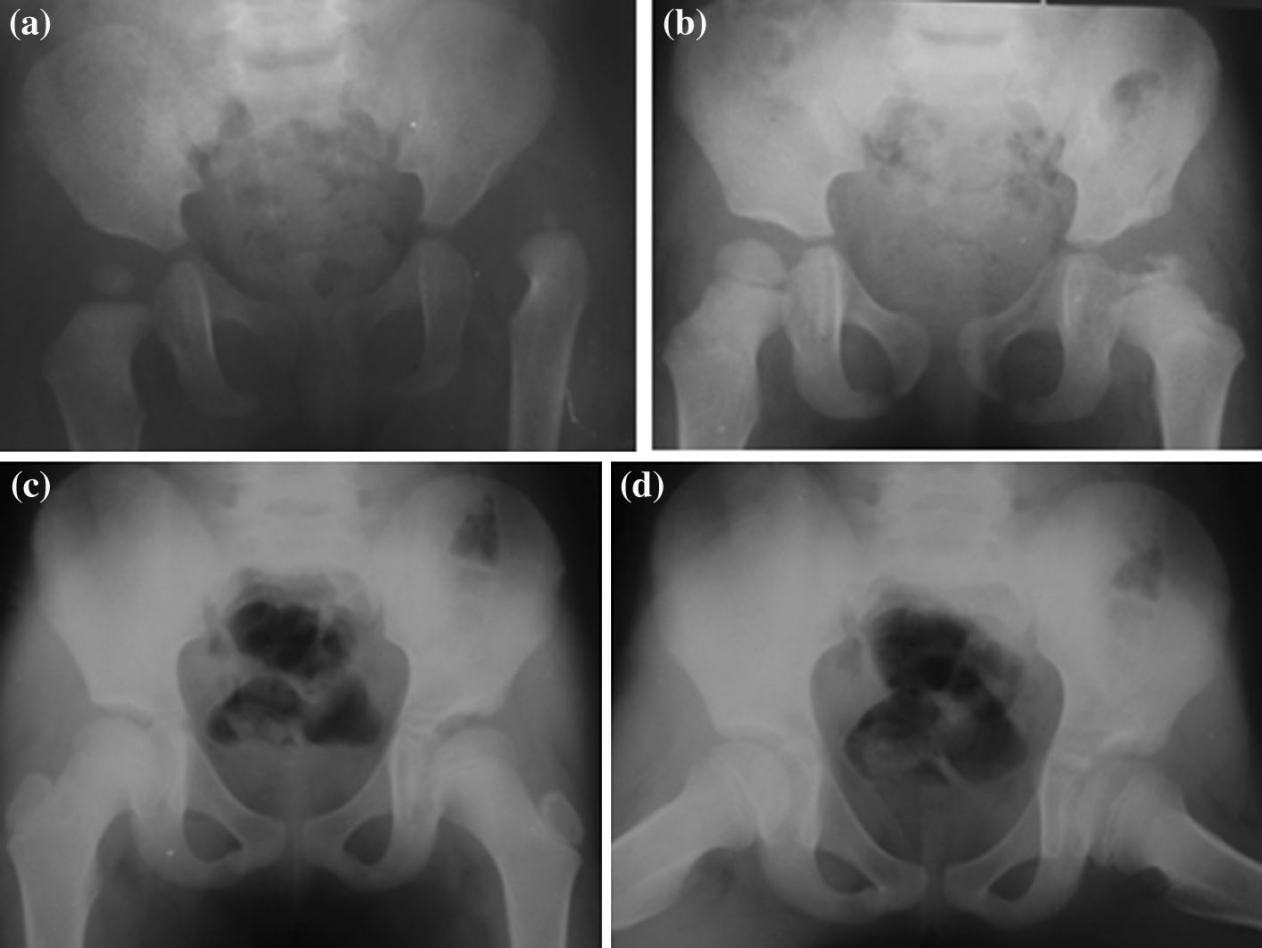
**a** Pelvis X-ray of 18-month-old girl, **b** 2-year post-operative pelvis X-ray shows type-1 avascular necrosis (AVN), **c**, **d** anteroposterior pelvis X-rag (**c**) and frog-leg X-ray (**d**) at 9 years of age shows no sign or sequel of AVN

The early outcomes from open reduction by the modified medial approach in the 55 hips were as follows: 43 hips (78.2 %) scored 6 (excellent), eight hips (14.5 %) scored 5 (good), and four hips (7.3 %) scored 4 (fair–plus). Unsatisfactory results were obtained due to the acetabular angle of Sharp and ATD values in a patient who underwent right hip surgery at 15 months of age, another who underwent the same surgery at 21 months of age, and in a patient who had a bilateral operation at 21 months of age due to a secondary intervention. Excellent and good (satisfactory) results were obtained in 92.7 % of the hips (Fig. 10), and fair–plus (unsatisfactory) results were obtained in 7.3 %.

Of 55 hips treated, type-1 temporary AVN was found in two patients (3.6 %); these lesions had disappeared completely from the last check-up graphs of these patients. Only one of these patients required a secondary osseous intervention. This patient had undergone open reduction using a bilateral modified medial approach due to bilateral hip dysplasia at 21 months of age. During the follow-up of this patient, the acetabular cover was thought to be insufficient, and a bilateral iliac (Salter innominate osteotomy) and femoral osteotomy (femoral shortening and derotation) were performed in different sessions 2 years after the first operation. None of the other patients required secondary osseous intervention. The rate of secondary osseous intervention was 3.6 %.

In children aged 11–18 months, postoperative treatment was concluded with plaster casting for an average of 3 months, followed by bracing with the modified Ilfeld device for 4 months and then by bracing with the device at night only for approximately 2 additional months. After sufficient acetabular coverage had been achieved, the device was removed and follow-up visits were conducted periodically. However, with increasing patient age, this period of time became longer. Children aged 18–28 months were treated by plaster casting for 3.5 months, the modified Ilfeld device was used for 6 months, and the device was used at night only for 2 additional months (Fig. 9). The treatment period with the modified Ilfeld device was close to 1 year in some patients. This treatment was maintained until the acetabular angle of Sharp reached a normal value. Despite such long periods of plaster casting and bracing, none of the hips were functionally restricted.

None of the other complications, such as infection, redislocation, or resubluxation, were seen in the operated patients.

## Discussion

Open reduction of DDH by the medial approach is one of the most effective surgical treatment methods during early childhood [7]. Two main medial intervention methods are currently being applied: Ludloff’s method (anteromedial) and Ferguson’s method (posteromedial) [14]. Medial intervention was first described by Ludloff [8] but it was not adopted for many years, becoming popular in the early 1970s following modifications by Mau et al. [9] and later by Ferguson [10]. In the original technique, Ludloff [8] used a skin incision of about 15 cm that begins with the Poupart ligament and passes the lateral border of the adductor longus toward the femoral axis, and the femoral vessel nerve pack is moved laterally with the help of a retractor. Ludloff [8] reached the capsule and iliopsoas tendon using the interval on the anterolateral border of the adductor longus. This method was modified to an anteromedial intervention by Mau et al. [9] who performed a longitudinal skin incision of about 5 cm that started from the insertion point of the hip adductor muscles in parallel with the adductor muscles and reached the capsule through the interval between the iliopsoas and pectineus muscles. The deep branch of the medial circumflex artery in front of the capsule was tied or retracted [9]. Ferguson [10] reported that he used a longitudinal incision that began with the inguinal crease and continued along the posterior side of the adductor longus. He reached the iliopsoas and capsule using the interval in the posteromedial of the adductor longus. Therefore, there was no need to reveal the femoral vessel nerve pack [10].

Weinstein and Ponseti reported that they applied a similar method to the one described by Ludloff [8], but they introduced a transverse skin incision [15]. These authors stated that they gently excluded the femoral vessel nerve pack together with the iliopsoas toward the lateral and found the obturator nerve, while trying to protect the branches of the medial circumflex artery [15]. However, they mostly (18/20 hips) had to tie the medial circumflex artery [15]. They reached the capsule through the pectineus and femoral vessel nerve pack using the interval also used by Mau et al. [9]. In Ludloff’s [8] method and all of its modifications, it is essential to reveal the femoral vessel nerve pack and exclude it gently and laterally with the help of a retractor in order to try to protect the medial circumflex artery intercrossing the operative field; the medial circumflex artery has even to be tied in some cases to reveal the obturator nerve and protect it as well [8, 9, 15].

Bicimoglu et al. [6] have described limited medial intervention. In their technique, adductor longus and iliopsoas tenotomy are performed using Ferguson’s posteromedial entrance. The hip is then reduced, and arthrography is conducted without opening the capsule. If the arthrogram shows Tonnis grade 1, the wound is closed; If it is Tonnis grade 2 or 3, the inferomedial capsule is opened, the forms preventing the reduction are removed, and the hip is reduced and immobilized in a plaster cast in the human position [6, 16].

In both Ferguson’s modification and the limited medial intervention of Bicimoglu et al. [6], the modification is to reach the capsule and iliopsoas through the same interval. These techniques also require that the medial circumflex artery be identified intercrossing the operative field and protected to reveal the obturator nerve to protect it as well [6, 10].

The challenging parts of an open reduction by the medial approach are to reveal the femoral vessel nerve pack and subsequently exclude it and also to find the medial circumflex artery and obturator nerve and protect them. Thus, a possibility of neurovascular damage always exists during these procedures. In particular, medial circumflex artery damage might result in AVN, which is one of the most feared complications during treatment.

Our analysis of open surgery methods by the medial approach showed that all of the methods reach the capsule and iliopsoas tendon through the same interval. When a common interval is used to reach these two locations, all procedures require that the medial circumflex artery be tied or excluded [6, 8–10, 15], and most require exclusion of the femoral vessel nerve pack [8, 9, 15]. Some techniques require revealing the obturator nerve [6, 10, 15]. In the technique we developed, a relatively smaller skin incision (3 cm) is used, and the capsule and iliopsoas tendon are reached through two different intervals. To reach the capsule, the interval in front of the pectineus is used in a similar way as in the method described by Mau et al. [9] and Weinstein and Ponseti [15]. The difference is that the femoral vessel nerve pack is not opened in our technique. We use Ferguson’s interval to locate the iliopsoas tendon [10]. The small trochanter should definitely be palpated here. We reach the iliopsoas only through this interval and do not open the capsule. Therefore, we have essentially minimized the interval to be cut. If a mistake occurs, and the iliopsoas is reached proximally through the small trochanter, the risk of injuring circumflex veins is present. In short, we use a smaller skin incision (3 cm) in our modified medial approach, the femoral vessel nerve pack is not opened, the circumflex veins are kept away from the operative field, and the operation is performed some distance from the obturator nerve.

Some authors have argued that open reduction by the medial approach is helpful [6, 9, 16], whereas others have reported that it leads to problems and high rates of AVN [17, 18]. The rate of AVN, which is the main complication of the medial approach, has been reported to vary from 0 to 67 %. The rate of dislocation is between 0 and 23 %, and the rate of the secondary operations varies between 0 and 53 % [6, 9, 10, 16–22]. Among our 41 patients (55 hips) we witnessed type-1 temporal AVN in only two patients (3.6 %), and the lesions ultimately disappeared completely in these patients. We consider that operating far from the circumflex artery using our technique had a great impact on the very low rate of AVN observed in our patients at the early and middle stages. Only one patient (3.6 %) required a secondary intervention due to the insufficiency in the acetabular cover on both hips. Redislocation was not observed in any of our patients.

A common consensus is that the upper age limit for open reduction-only treatment without osseous intervention is 18 months. However, how long the spontaneous remission potential of osseous acetabular dysplasia in a replaced hip lasts is controversial. During the 1960s, under the leadership of R.B. Salter, the suggestion was made that osseous dysplasia of the acetabulum could not be improved after 18 months. Subsequent studies found that this improvement could continue until 5 and even 8 years of age [23]. It has since been shown that medial open reductions can be performed securely until 24 months [20, 21, 24]. In agreement with these latter studies, we obtained satisfactory results from the majority of patients in our study. More than half of our patients were >18 months, and the incidence of a secondary osteotomy was very low.

The use of rigid abduction is usually reported following plaster casting [5, 6, 16]. The modified Ilfeld device used in our study enables hips to move in the direction of flexion–extension between 80° and 130°, and both hips can perform adduction and abduction movements in the safe zone symmetrically and at the same time. We believe that these movements of the hip have a positive impact on the functional capacity following use of this device. We also suggest that the device we use plays an important role as none of our patients had a functional restriction.

Radiology is mostly assessed according to the Severin classification [11]. However, this classification system is not reliable because all data except the CE angle are subjective, and the femoral part of the joint is neglected. For example, in a type-3 AVN hip, femoral head roundness is not yet deformed, the femoral length is too short, and the coxa breva that develops is grade I according to the Severin classification [7]. Omeroglu et al. introduced a new radiologic classification system based on all of these factors [11], and we therefore, we evaluated radiological outcomes in our study according to these new evaluation criteria [11].

There are a number of limitations to our study due to the short follow-up period. The major problem with the conventional medial approach is the prevalence of late Kalamchi–McEwen type-II AVN, which may not present itself until the age of 10 years. This may have resulted in the lower rate of AVN in our study population.

In conclusion, we obtained excellent or good results in 92.7 % of the patients treated with our modified medial intervention due to DDH. We believe that treatment for DDH using this modified medial approach during early childhood is an effective and reliable method with low complication rates and with which we can achieve great success in radiologic and clinical outcomes after a minimum 5-year follow-up period.
